# Sphingolipid homeostasis and dysregulation in liver function and disease

**DOI:** 10.1093/lifemeta/loag011

**Published:** 2026-04-24

**Authors:** Jianfeng Lan, Zhixiong Pan, Wei Dong, Junnan Wang, Chong Zhang, Yong Zhang, Yepeng Wu, Junfei Jin

**Affiliations:** Guangxi Key Laboratory of Molecular Medicine in Liver Injury and Repair, The First Affiliated Hospital of Guilin Medical University, Guilin, Guangxi 541001, China; Guangxi Health Commission Key Laboratory of Basic Research in Sphingolipid Metabolism Related Diseases, The First Affiliated Hospital of Guilin Medical University, Guilin, Guangxi 541001, China; China-USA Lipids in Health and Disease Research Center, Guilin Medical University, Guilin, Guangxi 541001, China; Laboratory of Hepatobiliary and Pancreatic Surgery, The First Affiliated Hospital of Guilin Medical University, Guilin, Guangxi 541001, China; Guangxi Key Laboratory of Molecular Medicine in Liver Injury and Repair, The First Affiliated Hospital of Guilin Medical University, Guilin, Guangxi 541001, China; Guangxi Health Commission Key Laboratory of Basic Research in Sphingolipid Metabolism Related Diseases, The First Affiliated Hospital of Guilin Medical University, Guilin, Guangxi 541001, China; China-USA Lipids in Health and Disease Research Center, Guilin Medical University, Guilin, Guangxi 541001, China; Laboratory of Hepatobiliary and Pancreatic Surgery, The First Affiliated Hospital of Guilin Medical University, Guilin, Guangxi 541001, China; Guangxi Key Laboratory of Molecular Medicine in Liver Injury and Repair, The First Affiliated Hospital of Guilin Medical University, Guilin, Guangxi 541001, China; Guangxi Health Commission Key Laboratory of Basic Research in Sphingolipid Metabolism Related Diseases, The First Affiliated Hospital of Guilin Medical University, Guilin, Guangxi 541001, China; China-USA Lipids in Health and Disease Research Center, Guilin Medical University, Guilin, Guangxi 541001, China; Laboratory of Hepatobiliary and Pancreatic Surgery, The First Affiliated Hospital of Guilin Medical University, Guilin, Guangxi 541001, China; Guangxi Key Laboratory of Molecular Medicine in Liver Injury and Repair, The First Affiliated Hospital of Guilin Medical University, Guilin, Guangxi 541001, China; Guangxi Health Commission Key Laboratory of Basic Research in Sphingolipid Metabolism Related Diseases, The First Affiliated Hospital of Guilin Medical University, Guilin, Guangxi 541001, China; China-USA Lipids in Health and Disease Research Center, Guilin Medical University, Guilin, Guangxi 541001, China; Laboratory of Hepatobiliary and Pancreatic Surgery, The First Affiliated Hospital of Guilin Medical University, Guilin, Guangxi 541001, China; Guangxi Key Laboratory of Molecular Medicine in Liver Injury and Repair, The First Affiliated Hospital of Guilin Medical University, Guilin, Guangxi 541001, China; Guangxi Health Commission Key Laboratory of Basic Research in Sphingolipid Metabolism Related Diseases, The First Affiliated Hospital of Guilin Medical University, Guilin, Guangxi 541001, China; China-USA Lipids in Health and Disease Research Center, Guilin Medical University, Guilin, Guangxi 541001, China; Laboratory of Hepatobiliary and Pancreatic Surgery, The First Affiliated Hospital of Guilin Medical University, Guilin, Guangxi 541001, China; Guangxi Key Laboratory of Molecular Medicine in Liver Injury and Repair, The First Affiliated Hospital of Guilin Medical University, Guilin, Guangxi 541001, China; Guangxi Health Commission Key Laboratory of Basic Research in Sphingolipid Metabolism Related Diseases, The First Affiliated Hospital of Guilin Medical University, Guilin, Guangxi 541001, China; China-USA Lipids in Health and Disease Research Center, Guilin Medical University, Guilin, Guangxi 541001, China; Laboratory of Hepatobiliary and Pancreatic Surgery, The First Affiliated Hospital of Guilin Medical University, Guilin, Guangxi 541001, China; Guangxi Key Laboratory of Molecular Medicine in Liver Injury and Repair, The First Affiliated Hospital of Guilin Medical University, Guilin, Guangxi 541001, China; Guangxi Health Commission Key Laboratory of Basic Research in Sphingolipid Metabolism Related Diseases, The First Affiliated Hospital of Guilin Medical University, Guilin, Guangxi 541001, China; China-USA Lipids in Health and Disease Research Center, Guilin Medical University, Guilin, Guangxi 541001, China; Laboratory of Hepatobiliary and Pancreatic Surgery, The First Affiliated Hospital of Guilin Medical University, Guilin, Guangxi 541001, China; Guangxi Key Laboratory of Molecular Medicine in Liver Injury and Repair, The First Affiliated Hospital of Guilin Medical University, Guilin, Guangxi 541001, China; Guangxi Health Commission Key Laboratory of Basic Research in Sphingolipid Metabolism Related Diseases, The First Affiliated Hospital of Guilin Medical University, Guilin, Guangxi 541001, China; China-USA Lipids in Health and Disease Research Center, Guilin Medical University, Guilin, Guangxi 541001, China; Laboratory of Hepatobiliary and Pancreatic Surgery, The First Affiliated Hospital of Guilin Medical University, Guilin, Guangxi 541001, China

**Keywords:** sphingolipids, chronic liver disease, metabolic dysfunction-associated steatohepatitis, liver fibrosis, hepatocellular carcinoma, biomarkers

## Abstract

Sphingolipids regulate hepatic lipid homeostasis, cell survival, inflammation, and tissue repair. In the healthy liver, balanced *de novo* sphingolipid synthesis, salvage pathways, and sphingosine-1-phosphate (S1P)-related signals maintain metabolic flexibility, endothelial integrity, and immune quiescence. Dysregulation of sphingolipid metabolism drives the initiation and progression of chronic liver diseases. In metabolic dysfunction-associated steatohepatitis, the acyl chain length-specific remodeling of dihydroceramides and ceramides, together with increased neutral sphingomyelinase activity, triggers lipotoxic stress, abnormal anabolic signal transduction, and hepatic lobule inflammation. Liver fibrosis involves reprogramming of the hepatic stellate cell S1P receptor signaling from regenerative toward profibrotic pathways. In hepatocellular carcinoma, tumor cells utilize sphingolipid metabolism to promote angiogenesis, evade immune surveillance, and develop therapeutic resistance. Sphingolipid remodeling in viral hepatitis links viral persistence to distinct circulating lipid signatures that correlate with disease severity and prognosis. Importantly, multiple nodes in the sphingolipid network and their downstream effectors are emerging as therapeutic targets. Promising preclinical strategies include liver-targeted small interfering RNA against key biosynthetic enzymes, selective modulation of sphingolipid receptors, and nanoliposomal formulations of bioactive ceramides. To enable clinical translation, innovative approaches are being developed to overcome key challenges in delivery, specificity, and safety. Overall, this review integrates recent mechanistic insights, emphasizing that sphingolipids act as central regulators of liver pathophysiology and are also important biomarkers and therapeutic targets in chronic liver diseases.

## Introduction

**Figure 1:**
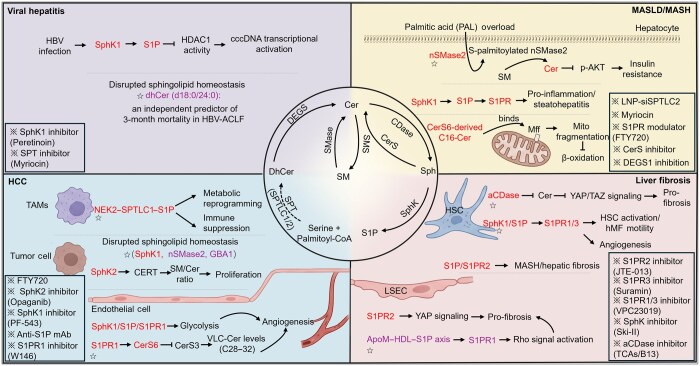
The sphingolipid metabolic hub links pathological remodeling and therapeutic opportunities in chronic liver diseases. The figure illustrates major sphingolipid metabolic changes and potential therapeutic targets across liver diseases, including viral hepatitis, MASLD/MASH, liver fibrosis, and hepatocellular carcinoma (HCC). SM, sphingomyelin; Cer, ceramide; DhCer, dihydroceramide; SPT, serine palmitoyltransferase; SphK, sphingosine kinase; S1P, sphingosine-1-phosphate; S1PR, S1P receptor; HSC, hepatic stellate cell; LSECs, liver sinusoidal endothelial cells; LNP, lipid nanoparticle; CERT, ceramide transfer protein; TCAs, tricyclic antidepressants; hMF, hepatic myofibroblast; TAM, tumor-associated macrophage; HDAC1, histone deacetylase 1; nSMase2, neutral sphingomyelinase-2; HDL, high-density lipoprotein; ApoM, apolipoprotein M; YAP, Yes-associated protein; TAZ, transcriptional coactivator with PDZ-binding motif; siRNA, small interfering RNA; VLC, very long chain ceramide; ☆, pathways or nodes directly supported by human clinical data; Red, pathological upregulation; Purple, pathological downregulation; ※, therapeutic intervention.

The liver plays a central role in maintaining lipid homeostasis through coordinated metabolic pathways, and sphingolipids are now recognized as key regulatory nodes in this network [1]. In addition to the well-known structural functions in cell membranes, sphingolipids can also serve as signal mediators to affect hepatocyte metabolism, support hepatic sinusoidal endothelial function, regulate intrahepatic immune responses, and promote tissue repair [2–4]. *De novo* sphingolipid synthesis initiates in the endoplasmic reticulum (ER), where serine palmitoyltransferase (SPT), an ER-resident enzyme complex with an SPT long chain base subunit 1 (SPTLC1)-SPTLC2 catalytic core, condenses serine and palmitoyl-CoA. This product is rapidly reduced to dihydrosphingosine, subsequently acylated by one of six ceramide synthases (CerS1 − 6), and finally desaturated by dihydroceramide desaturase 1 (DEGS1) to generate ceramides [4, 5]. Ceramides are then converted to sphingomyelin (SM) and complex glycosphingolipids. In parallel, sphingomyelinases generate ceramide via the salvage pathway, ceramidases liberate sphingosine, and sphingosine kinases 1 and 2 (SphK1/2) phosphorylate sphingosine to produce sphingosine-1-phosphate (S1P) (Fig. 1). S1P is irreversibly degraded by S1P lyase or exported to activate five S1P receptors (S1PR1 − 5), which are classified as G protein-coupled receptors, thereby coupling intracellular lipid metabolism via autocrine and paracrine signaling [6].

The sphingolipid pathway dynamically responds to metabolic cues. Lipotoxicity and oxidative stress upregulate SPT and specific CerS isoforms, thus promoting ceramide accumulation [1, 7]. In contrast, insulin and growth factors stimulate SphK-mediated S1P production under certain conditions [8]. This pathological imba­lance in sphingolipid metabolism, characterized by the accumulation of lipotoxic ceramides at the expense of pro-regenerative S1P signaling, promotes the progression of various liver diseases. Specific ceramide and dihydroceramide species accumulate in metabolic dysfunction-associated steatotic liver disease (MASLD) and metabolic dysfunction-associated steatohepatitis (MASH), contributing to lipotoxicity, inflammation, and fibrosis [9, 10]. Altered S1PR signaling can either support liver regeneration or exacerbate hepatic injury, depending on the cell type and S1PR subtype [6, 11]. In hepatocellular carcinoma (HCC), tumor cells reprogram the sphingolipid pathway to sustain angiogenesis and immune evasion [12–14]. Viral hepatitis has also been linked to sphingolipid remodeling, although the underlying mechanisms remain incompletely understood [15–18].

This review integrates recent mechanistic insights to explain how the sphingolipid network regulates liver pathophysiology. The literature reviewed herein was selected based on its mechanistic significance and translational impact, bridging fundamental biochemistry with clinical evidence to identify new therapeutic targets. We describe how the ceramide/dihydroceramide balance is coupled to cell-type-specific S1P−S1PR signaling among hepa­tocytes, stellate cells, and immune cells to determine whether the liver maintains homeostasis or progresses toward disease. Specifically, we analyze how dysregulation of these lipid axes drives the development of MASH, promotes fibrotic remodeling, and supports the growth and immune evasion of HCC. Finally, we evaluate the translational potential of sphingolipid-based biomarkers and therapies, while delineating the major barriers and emerging strategies, ranging from physical matrix disruption to biological engineering, to restore hepatic homeostasis and achieve clinical success.

## Characteristics of sphingolipid metabolism in the liver

Hepatic sphingolipid metabolism is perhaps best regarded as a highly compartmentalized process, with distinct patterns of enzyme and S1PR expression across different liver cell types rather than a uniform pathway in hepatocytes alone [19]. This cell-type-specific arrangement largely determines how changes in the ceramide−S1P balance affect downstream signaling, including metabolic regulation, inflammation, and repair responses [11]. It is worth noting that S1PR1 serves as the canonical mediator of endothelial cell homeostasis and regeneration within these cellular compartments. The role of S1PR2 is more variable, being strongly influenced by the surrounding microenvironment and specific types of damage [11, 20–23].

At the biochemical level, the precise composition of sphingolipids plays a crucial role in regulating signal transduction. For example, the 4,5-trans double bond introduced by DEGS1 endows ceramides with unique biophysical properties and signaling behaviors. This modification underlies the chain-length- and saturation-dependent effects observed in metabolic liver disease [24]. Upstream transcriptional programs further regulate the production of sphingolipids in the liver. ER stress triggers the unfolded protein response (UPR). During this process, the spliced form of X-box binding protein 1 (sXBP1) regulates the transcription of SPT, the rate-limiting enzyme in *de novo* sphingolipid synthesis [25]. Under conditions of nutrient overload or persistent inflammation, this mechanism may promote ceramide synthesis and amplify stress-related pathways. Importantly, single-cell transcriptomics and lipidomics studies exhibit clear liver metabolic pathways, which display marked zonal specificity and are restricted to distinct cell populations, rather than being uniformly distributed across liver lobules [26–29]. Cell-type-specific enzyme expression, receptor distribution, and spatial specificity together constitute a signal framework, which underlies both homeostatic maintenance and disease reprogramming.

## Physiological functions of sphingolipids in liver homeostasis

S1P is the main pro-survival and pro-regenerative signal in the liver [20, 21], although S1PR expression in hepatocytes is relatively low. The biological effect of S1P signaling mainly depends on its metabolic source, especially the two isoenzymes SphK1 and SphK2 [30, 31]. Although both are highly expressed in the liver, they show different subcellular compartmentalization, resulting in functionally distinct S1P pools [32, 33]. SphK1 is primarily localized in the cytosol and reduces the ceramide pool by diverting sphingosine toward the degradation pathway, generally promoting cell survival. However, under pathological conditions such as acute liver failure, hepatic SphK1 can exacerbate injury by promoting ER stress and mitochondrial permeability transition [34]. Alternatively, SphK2 is the major isoform in the liver and localizes to the nucleus, ER, and mitochondria [32]. Under metabolic stress, SphK2 is transcriptionally upregulated to enhance fatty acid oxidation, ameliorating steatosis and insulin resistance [35].

Beyond intracellular compartmentalization, the systemic and paracrine signaling of S1P is further regulated by its specific cha­perone system. Extracellular S1P is mainly carried in circulation by high-density lipoprotein (HDL)-associated apolipoprotein M (ApoM), so that it can be delivered to endothelial cells as a biased agonist [36]. Although ApoM is primarily synthesized by hepatocytes, the S1P cargo that it carries mainly originates from extrahepatic sources. In the liver, this ApoM-HDL-S1P axis activates S1PR1 on liver sinusoidal endothelial cells (LSECs) and promotes rege­nerative remodeling by inhibiting the pro-fibrotic Rho signaling pathway [20] (Fig. 1). These findings highlight that the ApoM-HDL-S1P axis maintains endothelial homeostasis, although its precise contribution to liver fibrosis and repair likely involves integration with additional signaling pathways.

As a key component of the plasma membrane (PM), SM faci­litates the formation of cholesterol-dependent raft domains through its specific sphingosine backbone and acyl chain interactions [37–39]. Single-molecule imaging studies demonstrate that SM molecules continuously and dynamically exchange between these ordered raft domains and the bulk membrane [39]. This structural action complements S1P signaling, which stabilizes the membrane domains and promotes molecular transport and stress sensing. Moreover, sphingolipid homeostasis suppresses non-parenchymal cell inflammation [40, 41]. During sepsis, hepatic macrophages, including Kupffer cells, govern inflammatory responses through the toll-like receptor 4/myeloid differentiation primary response 88/nuclear factor kappa B (TLR4/MyD88/NF-κB) axis. SphK1-mediated S1P production increases the S1P/ceramide ratio, which attenuates inflammatory cascades and preserves immune tolerance [42]. Under physiological conditions, the strictly regulated sphingolipid metabolism coordinates liver homeostasis and prevents tissue damage. Conversely, excessive ceramide accumulation or disrupted S1P signaling drives the progression of chronic liver diseases. These changes are thought to be linked to the initial steatosis, subsequent inflammatory reactions, and eventual fibrotic remodeling.

## Sphingolipid metabolism dysregulation across liver diseases

### Metabolic dysfunction-associated steatotic liver disease/metabolic dysfunction-associated steatohepatitis

Clinical investigations across independent patient cohorts have established a robust conclusion. MASLD/MASH features profound alterations in the hepatic and circulating sphingolipid profiles, rather than a simple global elevation [10, 43]. The hallmark of this alteration is an acyl-chain-length-specific imbalance. This pathological shift is marked by the enrichment of specific species subsets, including long-chain ceramide species (e.g., C16:0) and very-long-chain dihydroceramide species (e.g., C24:0 and C24:1) [9, 10]. These changes function to drive the sphingolipid rheostat toward a pro-inflammatory state [26].

Emerging evidence reveals that the pathological effects of sphingolipids depend heavily on subcellular location. Palmitate induces the palmitoylation-dependent translocation of neutral sphingomyelinase-2 (nSMase2, also known as SMPD3) to the PM, where it generates ceramide to directly impair protein kinase B (AKT) phosphorylation, thereby driving insulin resistance in steatotic hepatocytes [44] (Fig. 1). This establishes a cell-autonomous mechanism of insulin resistance by directly translating extracellular fatty acid abundance into defective intracellular signaling. Nevertheless, parallel lipotoxic mechanisms further aggravate hepatocellular injury, which is in turn exacerbated by mitochondrial dysfunction and the upregulation of six-transmembrane epithelial antigen of prostate 3 (STEAP3). Mechanistically, CerS6-derived C16-ceramides physically interact with the mitochondrial fission factor (Mff) [45]. Such binding promotes mitochondrial fragmentation and suppresses β-oxidation (Fig. 1). Concurrently, overexpression of STEAP3 increases palmitic acid/oleic acid-induced lipid deposition by coupling with the transforming growth factor (TGF)-beta-activated kinase 1 (TAK1)-mediated stress pathway to exacerbate hepatic steatosis [46]. The S1P axis is also dysregulated in MASLD/MASH [47]. Hepatic SphK1 expression and activity are often upregulated during pathogenesis, contributing to elevated S1P levels that modulate proinflammatory signaling [1, 4, 48] (Fig. 1). Specifically, S1P signaling through S1PR1 in myeloid cells directs the recruitment of monocyte-derived macrophages and activates hepatic inflammation in murine MASH [47]. These findings suggest that the sphingolipid network operates as a key regulatory platform integrating metabolic and stress signals. And the spatial compartmentalization indicates that MASH progression is less influenced by the total hepatic fat burden and more by spatially organized sphingolipid flux at organelle interfaces. Despite these advances, several limitations remain. The cellular origin of altered sphingolipid species in human patients remains unclear, particularly whether elevated C16-ceramides arise from hepatocyte synthesis. The rapid interconvertibility of bioactive sphingolipids also poses a daunting task for drug development.

### Liver fibrosis

Liver fibrosis is defined by excessive extracellular matrix (ECM) deposition mainly driven by activated hepatic stellate cells (HSCs). Upon activation, HSCs transdifferentiate into hepatic myofibroblasts (hMFs) that produce collagen and other fibrogenic factors [27, 49]. This fibrotic transition involves a profound reprogramming of the S1P−S1PR signaling axis, reflecting a fundamental disruption in sphingolipid homeostasis. Under chronic injury, the S1P rheostat diverges from its physiological role in regeneration toward profibrotic signaling pathways [27, 49, 50]. In the healthy liver, S1PR1 expressed in LSECs maintains vascular integrity and restrains pro-fibrotic Rho signaling [20]. During fibrosis, hepatic S1P levels are significantly elevated through upregulation of SphK1 regardless of the etiology of fibrosis [51]. This increase in S1P triggers a multifaceted profibrotic response, including HSC activation, ECM production, and directed migration. Analysis of human fibrotic specimens shows that hMFs markedly induce S1PR1 and S1PR3 expression, while S1PR2 expression often diminishes. S1P serves as a powerful stimulus that promotes the migration of human hMFs into injured hepatic regions. S1PR1 and S1PR3 primarily mediate this migratory effect, as pharmacological blockade or small interfering RNA (siRNA)-mediated silencing of these receptors significantly reduces cell motility. In contrast, S1PR2 signaling appears to inhibit this specific effect, suggesting that a complex, receptor-specific regulatory network governs cell recruitment during injury [51].

The S1P-S1PR3 pathway establishes a critical link between fibrosis and pathological angiogenesis. Within activated HSCs, S1P stimulation induces the expression of angiogenic markers via S1PR1 and S1PR3, which promotes vessel formation and further facilitates fibrotic progression [52]. However, elevated S1P levels in MASH livers predominantly promote fibrogenesis through S1PR2 signaling [23]. Correspondingly, activation of S1PR2 in LSECs promotes fibrotic progression through Yes-associated protein (YAP) signaling, reinforcing the structural remodeling of the liver niche [22]. This signaling network operates in tandem with ceramide, whose accumulation in hepatocytes promotes apoptosis and initiates injury [53]. However, the acid ceramidase (aCDase)−ceramide axis in HSCs functions as a molecular brake to restrain activation. Patients with advanced fibrosis exhibit upregulated aCDase in activated HSCs [54]. Inhibiting aCDase suppresses YAP/transcriptional coactivator with PDZ-binding motif (TAZ) sig­naling to reduce fibrosis in mice [54] (Fig. 1). These distinct roles are further underscored by lipidomics, which identifies SM (34:1) as a hallmark lipid specifically enriched within fibrotic regions [55].

In summary, sphingolipid dysregulation in liver fibrosis centers on HSC-specific S1PR reprogramming. Pharmacological or genetic interventions targeting this network demonstrate promising therapeutic potential in reversing established fibrosis [55]. A major challenge in fibrosis research is the functional duality of S1PRs [49, 50]. It is still difficult to accurately define the contribution of specific ceramide types in human clinical samples [53]. Finally, even the United States Food and Drug Administration (FDA)-approved non-invasive surrogates, such as vibration-controlled transient elastography (VCTE) and magnetic resonance elastography (MRE), struggle with significant sample variability [56, 57].

### Hepatocellular carcinoma

HCC is characterized by sphingolipid metabolic reprogramming, marked by upregulation of CerS5, which drives tumor progression [58], and downregulation of nSMase2, which functions as a tumor suppressor [12]. Notably, nSMase2-deficient mice develop spontaneous liver tumors with age, a process regulated by the expansion of cancer stem-like cells and the activation of the signal transducer and activator of transcription 3 (STAT3) signaling pathway [59]. Meanwhile, glycosphingolipid metabolism is frequently altered, as evidenced by glucosylceramidase beta 1 (GBA1) downregulation and the subsequent accumulation of glucosylceramide, which triggers aggressive signal through aberrant activation of the β-catenin pathway [13] (Fig. 1).

The SphK/S1P axis plays a key role in hepatocarcinogenesis by coordinating tumor progression across multiple cell types within the tumor microenvironment (TME). This is supported by *in vivo* evidence that loss of *Sphk1* significantly attenuates diethylnitrosamine (DEN)-induced hepatocarcinogenesis in mice [60]. SphK2 also contributes to non-alcoholic fatty liver disease (NAFLD)-associated HCC by upregulating ceramide transfer protein (CERT) to maintain a high SM-to-ceramide ratio, thereby supporting tumor cell proliferation [61]. In vascular endothelial cells, S1PR1 signaling induces the nuclear translocation of CerS6 to suppress CerS3 transcription. The resulting reduction in anti-angiogenic very-long-chain ceramides alleviates phosphatase and tensin homolog (PTEN) inhibition and activates the AKT/extracellular signal-regulated kinase (ERK) axis to drive vessel sprouting [14]. In addition, the SphK1/S1P axis promotes angiogenesis by stabilizing 6-phosphofructo-2-kinase/fructose-2,6-biphosphatase 3 (PFKFB3) to meet the energetic demands of nascent vessels [62]. Beyond angiogenesis, the meta­bolic reprogramming of tumor-associated macrophages (TAMs) creates an immunosuppressive microenvironment that facilitates immune evasion. The never in mitosis A (NIMA)-related kinase 2 (NEK2) exerts a critical influence on the activity of SPTLC1 to drive S1P biosynthesis within TAMs, promoting tumor progression and immunotherapy resistance [63] (Fig. 1).

Taken together, HCC restructures sphingolipid metabolism through mechanisms including reduced nSMase2 expression, altered glycosphingolipid turnover, and dysregulated SphK/S1P/S1PR signaling, to sustain angiogenesis and immune evasion. Nevertheless, metabolic redundancy between enzymes such as SphK1 and SphK2 often enables tumor cells to bypass single-target treatment. Tumor heterogeneity makes it difficult to identify universal biomarkers, and ensuring the effective delivery of bioactive modulators into large solid lesions remains a significant challenge.

### Viral hepatitis

Sphingolipid remodeling in viral hepatitis constitutes a strategic hijacking of host metabolic resources to promote viral entry, persistence, and immune escape [15]. Host sphingolipids not only provide structural components for the viral envelope, but also mediate the viral life cycle. In hepatitis B virus (HBV) infection, the SphK1−S1P axis functions as an epigenetic regulator. Virus-induced SphK1 upregulation and subsequent S1P accumulation inhibit histone deacetylase 1 (HDAC1) activity, maintaining HBV covalently closed circular DNA (cccDNA) in a transcriptionally active state. Peretinoin counteracts with this effect by suppressing the SphK1/S1P signaling axis [18]. This mechanism complements virus dependence on the *de novo* sphingolipid synthesis. Inhibition of SPT by myriocin enhances the antiviral efficacy of pegylated interferon [16], indicating that the sphingolipid flux creates a favorable environment for viral proliferation. Clinically, chronic HBV infection is associated with reduced levels of specific sphingolipid species, especially dhCer (d18:0/24:0), which serves as an independent predictor of 3-month mortality in patients with HBV-acute liver failure (ACLF) [17] (Fig. 1). Recent studies in ACLF models have demonstrated that increased level of dhCer (d18:0/24:0) reduces mortality and alleviates liver injury, suggesting that this specific metabolite may act as a protective factor rather than merely a biomarker [64]. However, the translational utility of these findings is constrained by the biological differences in systemic lipid meta­bolism between humanized murine models and the substantial heterogeneity of HBV patients. In addition, the mechanistic basis of lipid-associated prognostic markers remains incompletely understood.

## Sphingolipids as biomarkers and therapeutic targets in liver diseases

### Circulating sphingolipids as biomarkers for liver disease diagnosis and prognosis

In MASLD/MASH, lipidomic profiling reveals progressive altera­tions in sphingolipid metabolism during disease transition. A prominent feature is the increased SM hydrolysis driven by nSMase2, resulting in an elevated serum ceramide-to-SM ratio, which closely correlates with disease severity [3] (Fig. 1). Studies have consistently shown that the hepatic production and accumulation of dihydroceramides and glycosylated ceramides in adult patients across several independent cohorts are increased [10]. Interestingly, similar metabolic disorders also occur in pediatric MASLD, where the elevated levels of circulating saturated-chain ceramide are closely associated with reduced insulin sensiti­vity [65].

In addition to steatotic liver disease, circulating sphingolipids serve as reliable indicators of fibrotic progression and overall clinical risk. In patients with advanced chronic liver diseases, serum S1P levels gradually decrease with the deterioration of the disease stage and become independent predictors of short-term mortality in ACLF. These findings demonstrate that S1P is a criti­cal biomarker for disease severity and prognosis [66]. This systemic decline in S1P likely reflects impaired hepatic regenerative capacity and disrupted vascular integrity during the late stages of chronic injury.

Furthermore, specific sphingolipid profiles have been reported as potential diagnostic markers of HCC. Serum lipidomic analysis of HCC patients revealed that the levels of ceramide, dihydroceramide, and S1P are generally increased compared to individuals with cirrhosis alone. Among these altered species, C16-ceramide and S1P display the highest diagnostic accuracy in the studied cohorts [67]. This clinical observation is consistent with precli­nical findings where nSMase2 deficiency triggers a ­compensatory upregulation of CerS5, leading to a marked accumulation of C16-ceramide within tumor tissues [59]. This alignment between circulating lipid profiles and intratumoral metabolic reprogramming underscores the potential of sphingolipids as non-invasive tools for early cancer detection and patient stratification.

### Therapeutic potential targeting *de novo* ceramide synthesis and S1PR signaling in MASLD/MASH

Targeting sphingolipid pathways provides a mechanistically anchored strategy for MASH, as these lipids link hepatocyte lipotoxicity, inflammatory amplification, and fibrogenic progression [68]. Among the upstream metabolic nodes, the *de novo* ceramide synthesis pathway is attractive because it directly controls hepatic ceramide burden and downstream stress signaling. Pharmacological inhibition of serine SPT by myriocin prevents ceramide accumulation and reverses metabolic injury in experimental models [69]. This therapeutic concept is further validated by liver-directed lipid nanoparticle (LNP)-mediated delivery of siRNA against SPTLC2, which robustly reduces both hepatic and circulating ceramides and improves metabolic parameters, while simultaneously attenuating inflammation and fibrosis in dietary MASH models [70]. Meanwhile, DEGS1 suppression restores the dihydroceramide to ceramide balance and normalizes stress-associated sphingolipid species, thereby improving hepatic insulin signaling and steatosis [24, 26]. Inhibiting CerS6 with fumonisin B1 (FB1) effectively mitigates the C16-ceramide−mitochondrial fragmentation axis associated with hepatic insulin resistance and steatosis [45, 71]. In addition, pharmacologic mo­dulation of S1PR with FTY720/fingolimod signaling has shown significant efficacy in rodent MASH models, validating the functional role of the S1P axis in disease propagation [72] (Table 1).

**Table 1:** Sphingolipid-targeted therapeutic approaches for liver diseases.^a^

Therapeutic strategy	Key agents/targets	Mechanisms
**siRNA approaches**	LNP-siSPTLC2 (liver-targeted)	Ameliorating MASH by reducing hepatic/circulating ceramides [70] (validated by FDA-approved patisiran [73])
**S1PR modulators**	FTY720 (fingolimod)	MASH: attenuating steatosis via epigenetic regulation [72]
		HCC: suppressing tumorigenesis by disrupting CERS5 [58] and resensitizing TAMs [63]
	Etrasimod and ozanimod	Approved by the FDA for immune-mediated diseases; human safety data available [74, 75]
	S1PR1 inhibitor (W146)/lenvatinib	HCC: inhibiting pathological angiogenesis [14]
	S1PR1/3 inhibitor (VPC23019)	Liver fibrosis: significantly inhibiting angiogenesis [52]
	S1PR2 inhibitor (JTE-013)	Liver fibrosis: suppressing YAP-driven fibrogenesis [23]
**Biologics/active lipids**	Nanoliposome C6-ceramide	HCC: completed Phase I trial; favorable PK profile [76]; synergizing with anti-CTLA4 antibodies [77]
	Anti-S1P mAb	Reducing neovascularization and tumor invasion *in vitro* [78]
**Targeted inhibitors**	aCDase inhibitors (TCAs and B13)	Liver fibrosis: reversing established fibrosis by promoting YAP/TAZ degradation [54]
	SphK1 inhibitor (PF-543)	HCC: disrupting glycolytic energy supply to fuel tumor angiogenesis [62]
	SphK1/2 inhibitor (Ski-II)	Liver fibrosis: significantly reducing S1P to inhibit angiogenesis [52]
	SphK2 inhibitor (ABC294640)	HCC: rebalancing the anti-tumor ceramide pool and suppressing tumor (Phase II trial NCT02939807 withdrawn)
	SPT/CerS inhibitors (myriocin)	MASH: reversing metabolic injury [69]
	nSMase2 inhibitor (GW4869)	MASH: preventing lipotoxic inflammation [79]

a Abbreviations: aCDase, acid ceramidase; CerS, ceramide synthase; CTLA4, cytotoxic T-lymphocyte-associated protein 4; FB1, fumonisin B1; HCC, hepatocellular carcinoma; LNP, lipid nanoparticle; mAb, monoclonal antibody; MASH, metabolic dysfunction-associated steatohepatitis; nSMase2, neutral sphingomyelinase 2; PK, pharmacokinetics; S1PR, sphingosine-1-phosphate receptor; siRNA, small interfering RNA; SphK1/2, sphingosine kinase 1/2; SPT, serine palmitoyltransferase; SPTLC2, serine palmitoyltransferase long chain base subunit 2; TAM, tumor-associated macrophage; TCA, tricyclic antidepressant.

### Targeting SphK/S1P/S1PR signaling in fibrosis and hepatocellular carcinoma

The SphK/S1P/S1PR signaling represents an attractive but challenging therapeutic axis because receptor usage and responding cell types determine whether the clinical outcome is homeostatic or pathogenic [6, 11]. Effective intervention requires precise mo­dulation of this network to disrupt pathogenic signaling while preserving homeostatic functions. Pharmacological strategies in liver fibrosis focus on correcting the selective S1PR imbalance within activated HSCs [27, 49, 53, 80]. Interventions using the S1PR3 inhibitor suramin or siRNA-mediated silencing of S1P1 and S1P3 effectively block this S1P-induced hMF motility [50]. Evidence further shows that S1P reduction by SphK inhibitor Ski-II or administration of S1PR1/3 inhibitors such as VPC23019 signifi­cantly inhibits angiogenesis in fibrotic mice [52]. Additionally, targeted blockade of S1PR2 with JTE-013 reduces fibrosis in murine models by suppressing YAP-driven fibrogenesis [23]. In parallel, restoring the ceramide offers an alternative approach to stabilizing the sphingolipid rheostat. Inhibition of aCDase with tricyclic antidepressants (TCAs) or the small molecule B13 increases intracellular ceramide pools, thereby reducing fibrosis *in vivo* [54] (Table 1). These findings suggest that normalizing the sphingolipid rheostat within the fibrotic niche requires both the inhibition of profibrotic S1PRs and the maintenance of tumor-suppressive ceramide pools.

In HCC, pharmacological modulation of the sphingolipid network aims to disrupt angiogenesis and immune suppression. Strategies include neutralizing S1P with monoclonal antibodies or targeting its enzymatic source using selective SphK1 inhibitors such as PF-543 to limit nascent vessel sprouting [62, 78]. In parallel, genetic ablation or pharmacological inhibition of SphK2 with ABC294640 (also known as opaganib) offers a promising approach to rebalance the anti-tumor ceramide pool and suppress tumor growth in NAFLD-associated HCC [61]. To counteract immune evasion, targeting the TAM−S1P axis with FTY720 or NEK2 inhibitors represents a potent strategy to overcome anti-programmed cell death protein 1 (PD-1) therapy resistance [63]. Notably, FTY720 also suppresses HCC progression by indirectly disrupting the CerS5−lipophagy axis [58]. Regarding vascular control, selective pharmacological inhibition of S1PR1 by W146 or its downregulation by lenvatinib effectively impedes pathological angiogenesis and HCC progression [14] (Table 1). Underscoring the anti-angiogenic role of endogenous ceramides, the CerS inhibitor FB1 significantly facilitates tumor neovascularization and growth in experimental models [14]. Collectively, these interventions can be further augmented by the delivery of bioactive lipids, such as nanoliposomal C6-ceramide, which directly sensitizes HCC to immunotherapy [61, 63, 77].

## Clinical translation: therapeutic approaches, major barriers, and emerging strategies

### Sphingolipid-targeted therapeutic approaches

The translation of sphingolipid-targeted therapies has rapidly evolved from proof-of-concept models to advanced clinical eva­luations, leveraging diverse pharmacological platforms (Table 1).

- (i)LNP-siRNA approaches. Building on the clinical success of patisiran [73], the liver-targeted LNP platform has been successfully adapted for sphingolipid modulation. Specifically, LNP-mediated delivery of SPTLC2 siRNA has achieved robust knockdown of *de novo* ceramide synthesis in preclinical MASH models, offering a highly specific alternative to systemic chemical inhibitors [70]. Although clinical-stage siRNA candidates for sphingolipids are still emerging, the validated safety profile of hepatic LNP delivery significantly lowers the threshold for future clinical trials.
- (ii)S1PR modulators. The clinical landscape of S1P signaling is anchored by FDA-approved modulators such as FTY720, etrasimod, and ozanimod [74, 75, 81]. While originally indicated for multiple sclerosis or ulcerative colitis, these agents provide a wealth of human safety and pharmacokinetic data, facilitating their repositioning for liver disease. Beyond their established role in immune cell trafficking, next-generation S1PR modulators (e.g., W146) and multi-kinase inhibitors with S1PR-regulatory activity (e.g., lenvatinib) are currently being explored for their ability to remodel the TME and inhibit pathological angiogenesis in HCC [14].
- (iii)Biologics and bioactive lipids. Direct administration of bioactive sphingolipids has achieved a significant translational milestone with nanoliposome C6-ceramide. Having successfully completed Phase I trials in advanced solid tumors, this formulation demonstrated favorable pharmacokinetics and disease stabilization without dose-limiting toxicities [76]. In parallel, biologics such as neutralizing anti-S1P monoclonal antibodies are advancing as potential anti-angiogenic and anti-invasive therapies, targeting the extracellular S1P pool that drives malignancy [78].
- (iv)Targeted enzymes and kinase inhibitors. Small-molecule inhibitors targeting key metabolic nodes are undergoing rigo­rous validation. While upstream inhibitors such as myriocin (SPT inhibitor) and FB1 (CerS inhibitor) remain indispensable tools in preclinical studies [14, 69], clinical focus has shifted toward downstream S1PR modulators. Despite the recent withdrawal of the Phase II trial for ABC294640 (SphK2 inhibitor) in advanced HCC (NCT02939807), this setback has provided critical insights into patient stratification and the necessity of combinatorial regimens. Meanwhile, highly selective inhibitors such as PF-543 (SphK1 inhibitor) and various aCDase inhibitors are being refined to minimize systemic off-target effects while maximizing intrahepatic efficacy [54, 62].

### Major translational barriers

Despite compelling preclinical success, the clinical progression of sphingolipid-based therapies is impeded by a multifaceted landscape of biophysical and biological constraints:

(i) Delivery. The pathological changes, including LSEC defenestration in MASH, ECM deposition in fibrosis, and elevated interstitial pressure in HCC lesions, collectively create robust structural barriers that severely limit drug access [82–85]. (ii) Cell-type spe­cificity. The liver comprises multiple cell populations with distinct metabolic functions. Nanoparticles are frequently intercepted by Kupffer cells and TAMs, reducing payload delivery to target hepatocytes or HSCs [83]. (iii) Safety. Systemic administration of potent inhibitors (e.g., myriocin) can induce severe off-target effects, including systemic lymphopenia, bradycardia, and immunosuppression [86]. (iv) Patient heterogeneity. The complexity of human liver disease often dampens the efficacy of single-target therapies. A typical example is the recent termination of the Phase II clinical trial for BMS-986263 (HSP47 siRNA) [87]. Although it utilized an elegant vitamin A-coupled LNP [88] to specifically target activated HSCs, the trial (ClinicalTrials.gov ID: NCT03420768) demonstrated only modest antifibrotic efficacy in patients with advanced fibrosis. This outcome highlights the high therapeutic threshold required to reverse established fibrosis and suggests that disease stage and underlying biological heterogeneity may limit treatment responsiveness.

### Strategies to overcome clinical barriers

In order to overcome obstacles in delivery and ECM, researchers are utilizing ultrasound-guided microbubbles to transiently disrupt matrix barriers (sonoporation) [82] and deploying matrix-metalloproteinase (MMP)-functionalized “nanodrills” [89] to physically penetrate fibrotic tissue. To address cell-type specificity and the TME, researchers are developing engineered biological systems. For instance, the designer bacterium 1 (DB1) leverages interleukin-10 receptor (IL-10R) hysteresis to evade neutrophil phagocytosis, specifically target and reprogram TAMs, and effectively overcome dense tissue structures and aberrant blood vessels [90]. To reduce safety risks, physiologically based pharmacokinetic (PBPK) modeling is being employed for S1PR modulators such as etrasimod, providing a quantitative framework for precision dosing in patients with varying degrees of hepatic impairment to prevent the toxic accumulation of these agents [91]. Finally, to combat heterogeneity and enhance efficacy, combinatorial approaches are showing immense promise. Nanoliposomal C6-ceramide has been shown to synergize potently with anti-cytotoxic T-lymphocyte-associated protein 4 (CTLA4) antibodies [77], and targeting the NEK2−SPTLC1 axis in TAMs resensitizes resistant HCC to anti-PD-1 immunotherapy [63].

## Conclusions and perspective

Sphingolipid metabolism serves as a central regulator implicated in hepatic lipid homeostasis, inflammation, fibrosis, and tumorigenesis. In chronic liver diseases, the accumulation of specific ceramide and dihydroceramide, the enhancement of SM hydrolysis, and the reprogramming of S1PR signaling drive the progression from steatosis to MASH, fibrosis, and HCC. These pathophysiolo­gical shifts are characterized by spatially organized metabolic flux at organelle interfaces, indicating that sphingolipid metabolic disorders are key drivers of chronic liver diseases rather than merely secondary consequences.

These metabolic changes are reflected in circulating and tissue-specific signatures that reliably track disease severity across metabolic and viral etiologies. The identification of these lipid fingerprints offers a promising path for non-invasive diagnosis and patient stratification. Furthermore, the therapeutic potential of targeting the sphingolipid network is supported by strong preclinical evidence, with interventions against biosynthetic nodes or receptor pathways successfully reversing disease phenotypes. As this field moves beyond descriptive lipidomics, the current challenge lies in decoding the functional consequences of individual acyl chain lengths and their subcellular dynamics within the complex architecture of the human liver.

## Limitations of the study

Despite these exciting advances, there are still some key challenges, such as the limitations of spatial and cell-specific metabolic dynamics in the human liver, an incomplete understanding of the specific effects of acyl chains, and the need for liver- or cell-type-specific delivery to improve specificity and safety. Fortunately, the ongoing research on single-cell transcriptomics, stable isotope tracing, and tissue-specific gene silencing/degradation platforms is expected to solve these problems. An integrated system-level approach is poised to enable the clinical translation of validated sphingolipid biomarkers and mechanism-driven targeted therapies for the treatment of chronic liver diseases in the near future.

## Data Availability

Data sharing is not applicable to this article as no new datasets were generated or analyzed during the current study.

## References

[1] Green CD , Maceyka M, Cowart LA et al Sphingolipids in metabolic disease: the good, the bad, and the unknown. Cell Metab 2021;33:1293–306. 34233172 10.1016/j.cmet.2021.06.006 PMC8269961

[2] Hannun YA , Obeid LM. Principles of bioactive lipid signalling: lessons from sphingolipids. Nat Rev Mol Cell Biol 2008;9:139–50. 18216770 10.1038/nrm2329

[3] Jiang J , GaoY, WangJ et al Hepatic sphingomyelin phosphodiesterase 3 promotes steatohepatitis by disrupting membrane sphingolipid metabolism. Cell Metab 2025;37:1119–36 e13.40015281 10.1016/j.cmet.2025.01.016

[4] Kuo A , Hla T. Regulation of cellular and systemic sphingolipid homeostasis. Nat Rev Mol Cell Biol 2024;25:802–21. 38890457 10.1038/s41580-024-00742-y PMC12034107

[5] Hannun YA , Obeid LM. Sphingolipids and their metabolism in physiology and disease. Nat Rev Mol Cell Biol 2018;19:175–91. 29165427 10.1038/nrm.2017.107 PMC5902181

[6] Ogretmen B. Sphingolipid metabolism in cancer signalling and therapy. Nat Rev Cancer 2018;18:33–50. 29147025 10.1038/nrc.2017.96 PMC5818153

[7] Chaurasia B , Summers SA. Ceramides in metabolism: key lipotoxic players. Annu Rev Physiol 2021;83:303–30. 33158378 10.1146/annurev-physiol-031620-093815 PMC7905841

[8] Aji G , Huang Y, Ng ML et al Regulation of hepatic insulin signaling and glucose homeostasis by sphingosine kinase 2. Proc Natl Acad Sci USA 2020;117:24434–42. 32917816 10.1073/pnas.2007856117 PMC7533871

[9] Heymann CJF , Mak AL, Holleboom AG et al The plasma lipidome varies with the severity of metabolic dysfunction-associated steatotic liver disease. Lipids Health Dis 2024;23:402. 39696394 10.1186/s12944-024-02380-x PMC11653580

[10] Apostolopoulou M , Gordillo R, Koliaki C et al Specific hepatic sphingolipids relate to insulin resistance, oxidative stress, and inflammation in nonalcoholic steatohepatitis. Diabetes Care 2018;41:1235–43. 29602794 10.2337/dc17-1318

[11] Kleuser B. Divergent role of sphingosine 1-phosphate in liver health and disease. Int J Mol Sci 2018;19:722. 29510489 10.3390/ijms19030722 PMC5877583

[12] Revill K , Wang T, Lachenmayer A et al Genome-wide methylation analysis and epigenetic unmasking identify tumor suppressor genes in hepatocellular carcinoma. Gastroenterology 2013;145:1424–35.e25. 24012984 10.1053/j.gastro.2013.08.055 PMC3892430

[13] Qiu Z , Wang X, Yang Z et al GBA1-dependent membrane glucosylceramide reprogramming promotes liver cancer metastasis via activation of the Wnt/β-catenin signalling pathway. Cell Death Dis 2022;13:508. 35637196 10.1038/s41419-022-04968-6 PMC9151913

[14] Wang X , Qiu Z, Dong W et al S1PR1 induces metabolic reprogramming of ceramide in vascular endothelial cells, affecting hepatocellular carcinoma angiogenesis and progression. Cell Death Dis 2022;13:768. 36068200 10.1038/s41419-022-05210-z PMC9448762

[15] Dai J , Feng Y, Liao Y et al Virus infection and sphingolipid metabolism. Antiviral Res 2024;228:105942. 38908521 10.1016/j.antiviral.2024.105942

[16] Tatematsu K , Tanaka Y, Sugiyama M et al Host sphingolipid biosynthesis is a promising therapeutic target for the inhibition of hepatitis B virus replication. J Med Virol 2011;83:587–93. 21328371 10.1002/jmv.21970

[17] Qu F , Zheng SJ, Liu S et al Serum sphingolipids reflect the severity of chronic HBV infection and predict the mortality of HBV-acute-on-chronic liver failure. PLoS One 2014;9:e104988. 25136927 10.1371/journal.pone.0104988 PMC4138167

[18] Murai K , Shirasaki T, Honda M et al Peretinoin, an acyclic retinoid, inhibits hepatitis B virus replication by suppressing sphingosine metabolic pathway *in vitro*. Int J Mol Sci 2018;19:108. 29360739 10.3390/ijms19020108 PMC5855541

[19] Ma X , Huang T, Chen X et al Molecular mechanisms in liver repair and regeneration: from physiology to therapeutics. Signal Transduct Target Ther 2025;10:63. 39920130 10.1038/s41392-024-02104-8 PMC11806117

[20] Ding BS , Liu CH, Sun Y et al HDL activation of endothelial sphingosine-1-phosphate receptor-1 (S1PR1) promotes regeneration and suppresses fibrosis in the liver. JCI Insight 2016;1:e87058. 28018969 10.1172/jci.insight.87058 PMC5161208

[21] Ikeda H , Watanabe N, Ishii I et al Sphingosine 1-phosphate regulates regeneration and fibrosis after liver injury via sphingosine 1-phosphate receptor 2. J Lipid Res 2009;50:556–64. 18955732 10.1194/jlr.M800496-JLR200 PMC2638109

[22] Liao Y , Zhou C, Duan Y et al Liver sinusoidal endothelial S1pr2 regulates experimental liver fibrosis through YAP/TGF-β signaling pathway. FASEB J 2023;37:e22905. 37039817 10.1096/fj.202201954R

[23] Osawa Y , Kawai H, Nakashima K et al Sphingosine-1-phosphate promotes liver fibrosis in metabolic dysfunction-associated steatohepatitis. PLoS One 2024;19:e0303296. 38753743 10.1371/journal.pone.0303296 PMC11098361

[24] Chaurasia B , Tippetts TS, Mayoral Monibas R et al Targeting a ceramide double bond improves insulin resistance and hepatic steatosis. Science 2019;365:386–92. 31273070 10.1126/science.aav3722 PMC6787918

[25] Kim GT , Devi S, Sharma A et al Upregulation of the serine palmitoyltransferase subunit SPTLC2 by endoplasmic reticulum stress inhibits the hepatic insulin response. Exp Mol Med 2022;54:573–84. 35513574 10.1038/s12276-022-00766-4 PMC9166747

[26] Seubnooch P , Montani M, Dufour JF et al Spatial lipidomics reveals zone-specific hepatic lipid alteration and remodeling in metabolic dysfunction-associated steatohepatitis. J Lipid Res 2024;65:100599. 39032559 10.1016/j.jlr.2024.100599 PMC11388789

[27] Gonzalez-Fernandez B , Sanchez DI, Gonzalez-Gallego J et al Sphingosine 1-phosphate signaling as a target in hepatic fibrosis therapy. Front Pharmacol 2017;8:579. 28890699 10.3389/fphar.2017.00579 PMC5574909

[28] Otkur W , Zhang Y, Li Y et al Spatial multi-omics characterizes GPR35-relevant lipid metabolism signatures across liver zonation in MASLD. Life Metab 2024;3:loae021. 39873004 10.1093/lifemeta/loae021 PMC11748505

[29] Li Z , Luo G, Gan C et al Spatially resolved multi-omics of human metabolic dysfunction-associated steatotic liver disease. Nat Genet 2025;57:3112–25. 41286103 10.1038/s41588-025-02407-8 PMC12695644

[30] Diaz Escarcega R , McCullough LD, Tsvetkov AS. The functional role of sphingosine kinase 2. Front Mol Biosci 2021;8:683767. 34055895 10.3389/fmolb.2021.683767 PMC8160245

[31] Liu H , Sugiura M, Nava VE et al Molecular cloning and functional characterization of a novel mammalian sphingosine kinase type 2 isoform. J Biol Chem 2000;275:19513–20. 10751414 10.1074/jbc.M002759200

[32] Hait NC , Oskeritzian CA, Paugh SW et al Sphingosine kinases, sphingosine 1-phosphate, apoptosis and diseases. Biochim Biophys Acta 2006;1758:2016–26. 16996023 10.1016/j.bbamem.2006.08.007

[33] Maceyka M , Sankala H, Hait NC et al SphK1 and SphK2, sphingosine kinase isoenzymes with opposing functions in sphingolipid metabolism. J Biol Chem 2005;280:37118–29. 16118219 10.1074/jbc.M502207200

[34] Li L , Wang H, Zhang J et al SPHK1 deficiency protects mice from acetaminophen-induced ER stress and mitochondrial permeability transition. Cell Death Differ 2020;27:1924–37. 31827236 10.1038/s41418-019-0471-x PMC7244772

[35] Lee SY , Hong IK, Kim BR et al Activation of sphingosine kinase 2 by endoplasmic reticulum stress ameliorates hepatic steatosis and insulin resistance in mice. Hepatology 2015;62:135–46. 25808625 10.1002/hep.27804

[36] Galvani S , Sanson M, Blaho VA et al HDL-bound sphingosine 1-phosphate acts as a biased agonist for the endothelial cell receptor S1P1 to limit vascular inflammation. Sci Signal 2015;8:ra79. 26268607 10.1126/scisignal.aaa2581 PMC4768813

[37] Lingwood D , Simons K. Lipid rafts as a membrane-organizing principle. Science 2010;327:46–50. 20044567 10.1126/science.1174621

[38] Lin Q , London E. Ordered raft domains induced by outer leaflet sphingomyelin in cholesterol-rich asymmetric vesicles. Biophys J 2015;108:2212–22. 25954879 10.1016/j.bpj.2015.03.056 PMC4423047

[39] Kinoshita M , Suzuki KG, Matsumori N et al Raft-based sphingomyelin interactions revealed by new fluorescent sphingomyelin analogs. J Cell Biol 2017;216:1183–204. 28330937 10.1083/jcb.201607086 PMC5379944

[40] Lee M , Lee SY, Bae YS. Functional roles of sphingolipids in immunity and their implication in disease. Exp Mol Med 2023;55:1110–30. 37258585 10.1038/s12276-023-01018-9 PMC10318102

[41] Schwabe RF , BrennerDA. Hepatic stellate cells: balancing homeostasis, hepatoprotection and fibrogenesis in health and disease. Nat Rev Gastroenterol Hepatol 2025;22:481–99.40404839 10.1038/s41575-025-01068-6

[42] Wang B , Wu X, Cheng J et al Regulatory role of S1P and its receptors in sepsis-induced liver injury. Front Immunol 2025;16:1489015. 39935473 10.3389/fimmu.2025.1489015 PMC11811114

[43] Hajduch E , Lachkar F, Ferre P et al Roles of ceramides in non-alcoholic fatty liver disease. J Clin Med 2021;10:792. 33669443 10.3390/jcm10040792 PMC7920467

[44] El-Amouri S , Karakashian A, Bieberich E et al Regulated translocation of neutral sphingomyelinase-2 to the plasma membrane drives insulin resistance in steatotic hepatocytes. J Lipid Res 2023;64:100435. 37640282 10.1016/j.jlr.2023.100435 PMC10550728

[45] Hammerschmidt P , Ostkotte D, Nolte H et al CerS6-derived sphingolipids interact with Mff and promote mitochondrial fragmentation in obesity. Cell 2019;177:1536–52.e23. 31150623 10.1016/j.cell.2019.05.008

[46] Ding T , Chen S, Xiao W et al Six-transmembrane epithelial antigen of prostate 3 promotes hepatic insulin resistance and steatosis. J Lipid Res 2023;64:100318. 36495944 10.1016/j.jlr.2022.100318 PMC9823233

[47] Parthasarathy G , Venkatesan N, Sidhu GS et al Deletion of sphingosine 1-phosphate receptor 1 in myeloid cells reduces hepatic inflammatory macrophages and attenuates MASH. Hepatol Commun 2025;9:e0613. 39899672 10.1097/HC9.0000000000000613 PMC12333770

[48] Geng T , Sutter A, Harland MD et al SphK1 mediates hepatic inflammation in a mouse model of NASH induced by high saturated fat feeding and initiates proinflammatory signaling in hepatocytes. J Lipid Res 2015;56:2359–71. 26482537 10.1194/jlr.M063511 PMC4655991

[49] Delgado ME , Cardenas BI, Farran N et al Metabolic reprogramming of liver fibrosis. Cells 2021;10:3604. 34944111 10.3390/cells10123604 PMC8700241

[50] Ishay Y , Nachman D, Khoury T et al The role of the sphingolipid pathway in liver fibrosis: an emerging new potential target for novel therapies. Am J Physiol Cell Physiol 2020;318:C1055–64. 32130072 10.1152/ajpcell.00003.2020

[51] Li C , Zheng S, You H et al Sphingosine 1-phosphate (S1P)/S1P receptors are involved in human liver fibrosis by action on hepatic myofibroblasts motility. J Hepatol 2011;54:1205–13. 21145832 10.1016/j.jhep.2010.08.028

[52] Yang L , Yue S, Yang L et al Sphingosine kinase/sphingosine 1-phosphate (S1P)/S1P receptor axis is involved in liver fibrosis-associated angiogenesis. J Hepatol 2013;59:114–23. 23466305 10.1016/j.jhep.2013.02.021

[53] Park WJ , Song JH, Kim GT et al Ceramide and sphingosine 1-phosphate in liver diseases. Mol Cells 2020;43:419–30. 32392908 10.14348/molcells.2020.0054 PMC7264474

[54] Alsamman S , Christenson SA, Yu A et al Targeting acid ceramidase inhibits YAP/TAZ signaling to reduce fibrosis in mice. Sci Transl Med 2020;12:eaay8798. 32817366 10.1126/scitranslmed.aay8798 PMC7976849

[55] Gruevska A , Leslie J, Perpinan E et al Spatial lipidomics reveals sphingolipid metabolism as anti-fibrotic target in the liver. Metabolism 2025;168:156237. 40127860 10.1016/j.metabol.2025.156237

[56] Zhang Y , Lee HW, Lin H et al Head-to-head comparison between vibration-controlled transient elastography and histology in predicting liver-related events due to metabolic dysfunction-associated steatotic liver disease. Hepatology 2025. DOI: 10.1097/HEP.0000000000001658. 41452034

[57] Younossi ZM , Zelber-Sagi S, Lazarus JV et al Global consensus recommendations for metabolic dysfunction-associated steatotic liver disease and steatohepatitis. Gastroenterology 2025;169:1017–32.e2. 40222485 10.1053/j.gastro.2025.02.044

[58] Liu Q , Zhang X, Qi J et al Comprehensive profiling of lipid metabolic reprogramming expands precision medicine for HCC. Hepatology 2025;81:1164–80. 38899975 10.1097/HEP.0000000000000962 PMC11902616

[59] Zhong L , Kong JN, Dinkins MB et al Increased liver tumor formation in neutral sphingomyelinase-2-deficient mice. J Lipid Res 2018;59:795–804. 29567647 10.1194/jlr.M080879 PMC5928441

[60] Chen J , Qi Y, Zhao Y et al Deletion of sphingosine kinase 1 inhibits liver tumorigenesis in diethylnitrosamine-treated mice. Oncotarget 2018;9:15635–49. 29643998 10.18632/oncotarget.24583 PMC5884653

[61] Liu XT , Chung LH, Liu D et al Ablation of sphingosine kinase 2 suppresses fatty liver-associated hepatocellular carcinoma via downregulation of ceramide transfer protein. Oncogenesis 2022;11:67. 36333295 10.1038/s41389-022-00444-0 PMC9636415

[62] Liu XT , Huang Y, Liu D et al Targeting the SphK1/S1P/PFKFB3 axis suppresses hepatocellular carcinoma progression by disrupting glycolytic energy supply that drives tumor angiogenesis. J Transl Med 2024;22:43. 38200582 10.1186/s12967-023-04830-z PMC10782643

[63] Zhang X , Lao M, Sun K et al Sphingolipid synthesis in tumor-associated macrophages confers immunotherapy resistance in hepatocellular carcinoma. Sci Adv 2025;11:eadv0558. 40397754 10.1126/sciadv.adv0558 PMC12094245

[64] Li FF , Liu N, Liu W et al Role of dihydroceramides in the progression of acute-on-chronic liver failure in rats. Chin Med J (Engl) 2020;133:198–204. 31880746 10.1097/CM9.0000000000000601 PMC7028171

[65] Wasilewska N , Bobrus-Chociej A, Harasim-Symbor E et al Increased serum concentration of ceramides in obese children with nonalcoholic fatty liver disease. Lipids Health Dis 2018;17:216. 30208901 10.1186/s12944-018-0855-9 PMC6136227

[66] Mucke VT , Maria Schwarzkopf K, Thomas D et al Serum sphingosine-1-phosphate is decreased in patients with acute-on-chronic liver failure and predicts early mortality. Hepatol Commun 2020;4:1477–86. 33024917 10.1002/hep4.1561 PMC7527696

[67] Grammatikos G , Schoell N, Ferreiros N et al Serum sphingolipidomic analyses reveal an upregulation of C16-ceramide and sphingosine-1-phosphate in hepatocellular carcinoma. Oncotarget 2016;7:18095–105. 26933996 10.18632/oncotarget.7741 PMC4951274

[68] Xie Z , Li Y, Cheng L et al Potential therapeutic strategies for MASH: from preclinical to clinical development. Life Metab 2024;3:loae029. 39872142 10.1093/lifemeta/loae029 PMC11749562

[69] Yang RX , Pan Q, Liu XL et al Therapeutic effect and autophagy regulation of myriocin in nonalcoholic steatohepatitis. Lipids Health Dis 2019;18:179. 31639005 10.1186/s12944-019-1118-0 PMC6805575

[70] Yu X , Huang C, Evers M et al Targeted inhibition of hepatic *de novo* ceramide synthesis ameliorates MASH. Sci Adv 2025;11:eadx2681. 41004573 10.1126/sciadv.adx2681 PMC12466852

[71] Hammerschmidt P , Steculorum SM, Bandet CL et al CerS6-dependent ceramide synthesis in hypothalamic neurons promotes ER/mitochondrial stress and impairs glucose homeostasis in obese mice. Nat Commun 2023;14:7824. 38016943 10.1038/s41467-023-42595-7 PMC10684560

[72] Rohrbach TD , Asgharpour A, Maczis MA et al FTY720/fingolimod decreases hepatic steatosis and expression of fatty acid synthase in diet-induced nonalcoholic fatty liver disease in mice. J Lipid Res 2019;60:1311–22. 31110049 10.1194/jlr.M093799 PMC6602124

[73] Akinc A , Maier MA, Manoharan M et al The Onpattro story and the clinical translation of nanomedicines containing nucleic acid-based drugs. Nat Nanotechnol 2019;14:1084–7. 31802031 10.1038/s41565-019-0591-y

[74] Martinez-Molina C , Gonzalez-Suarez B. Etrasimod: modulating sphingosine-1-phosphate receptors to treat ulcerative colitis. J Clin Med 2025;14:3890. 40507651 10.3390/jcm14113890 PMC12155636

[75] Lamb YN. Ozanimod: first approval. Drugs 2020;80:841–8. 32385738 10.1007/s40265-020-01319-7

[76] Ciner A , Gourdin T, Davidson J et al A phase I study of the ceramide nanoliposome in patients with advanced solid tumors. Cancer Chemother Pharmacol 2024;93:23–9. 37736793 10.1007/s00280-023-04588-7 PMC10796569

[77] Qi X , Wu F, Kim SH et al Nanoliposome C6-ceramide in combination with anti-CTLA4 antibody improves anti-tumor immunity in hepatocellular cancer. FASEB J 2022;36:e22250. 35294071 10.1096/fj.202101707R PMC9297193

[78] Visentin B , Vekich JA, Sibbald BJ et al Validation of an anti-sphingosine-1-phosphate antibody as a potential therapeutic in reducing growth, invasion, and angiogenesis in multiple tumor lineages. Cancer Cell 2006;9:225–38. 16530706 10.1016/j.ccr.2006.02.023

[79] Al-Rashed F , Arefanian H, Madhoun AA et al Neutral sphingomyelinase 2 inhibition limits hepatic steatosis and inflammation. Cells 2024;13:463. 38474427 10.3390/cells13050463 PMC10931069

[80] Nojima H , Shimizu H, Murakami T et al Critical roles of the sphingolipid metabolic pathway in liver regeneration, hepatocellular carcinoma progression and therapy. Cancers (Basel) 2024;16:850. 38473211 10.3390/cancers16050850 PMC10931359

[81] Chun J , Brinkmann V. A mechanistically novel, first oral therapy for multiple sclerosis: the development of fingolimod (FTY720, Gilenya). Discov Med 2011;12:213–28. 21955849 PMC3694567

[82] Armillotta MG , Lizzi L, Massimi M. Nanoparticle-based systems for liver therapy: overcoming fibrosis and enhancing drug efficacy. World J Hepatol 2025;17:108810. 41179719 10.4254/wjh.v17.i10.108810 PMC12576705

[83] Sadauskas E , Wallin H, Stoltenberg M et al Kupffer cells are central in the removal of nanoparticles from the organism. Part Fibre Toxicol 2007;4:10. 17949501 10.1186/1743-8977-4-10 PMC2146996

[84] Szafranska K , Kruse LD, Holte CF et al The wHole story about fenestrations in LSEC. Front Physiol 2021;12:735573. 34588998 10.3389/fphys.2021.735573 PMC8473804

[85] Libutti SK , Tamarkin L, Nilubol N. Targeting the invincible barrier for drug delivery in solid cancers: interstitial fluid pressure. Oncotarget 2018;9:35723–5. 30515264 10.18632/oncotarget.26267 PMC6254664

[86] Yang X , Yan Y, Liu S et al Potential adverse events associated with sphingosine-1-phosphate (S1P) receptor modulators in patients with multiple sclerosis: an analysis of the FDA adverse event reporting system (FAERS) database. Front Pharmacol 2024;15:1376494. 38846098 10.3389/fphar.2024.1376494 PMC11153721

[87] Qosa H , de Oliveira C, Cizza G et al Pharmacokinetics, safety, and tolerability of BMS-986263, a lipid nanoparticle containing HSP47 siRNA, in participants with hepatic impairment. Clin Transl Sci 2023;16:1791–802. 37654022 10.1111/cts.13581 PMC10582666

[88] Sato Y , Murase K, Kato J et al Resolution of liver cirrhosis using vitamin A-coupled liposomes to deliver siRNA against a collagen-specific chaperone. Nat Biotechnol 2008;26:431–42. 18376398 10.1038/nbt1396

[89] Liu J , Liu J, Mu W et al Delivery strategy to enhance the therapeutic efficacy of liver fibrosis via nanoparticle drug delivery systems. ACS Nano 2024;18:20861–85. 39082637 10.1021/acsnano.4c02380

[90] Chang Z , Guo X, Li X et al Bacterial immunotherapy leveraging IL-10R hysteresis for both phagocytosis evasion and tumor immunity revitalization. Cell 2025;188:1842–57.e20. 40037354 10.1016/j.cell.2025.02.002

[91] Alasmari MS , Alqahtani F, Alasmari F et al Model-based dose selection of a sphingosine-1-phosphate modulator, etrasimod, in patients with various degrees of hepatic impairment. Pharmaceutics 2024;16:1540. 39771519 10.3390/pharmaceutics16121540 PMC11728834

